# Domain Shuffling between Vip3Aa and Vip3Ca: Chimera Stability and Insecticidal Activity against European, American, African, and Asian Pests

**DOI:** 10.3390/toxins12020099

**Published:** 2020-02-04

**Authors:** Joaquín Gomis-Cebolla, Rafael Ferreira dos Santos, Yueqin Wang, Javier Caballero, Primitivo Caballero, Kanglai He, Juan Luis Jurat-Fuentes, Juan Ferré

**Affiliations:** 1 ERI de Biotecnología y Biomedicina (BIOTECMED), Department of Genetics, Universitat de València, 46100-Burjassot, Spain; joaquin.gomis@uv.es; 2 Department of Entomology and Plant Pathology, University of Tennessee, Knoxville, TN 37996, USA; rferrei1@utk.edu (R.F.d.S.); jurat@utk.edu (J.L.J.-F.); 3 State Key Laboratory for Biology of Plant Diseases and Insect Pests, Institute of Plant Protection, Chinese Academy of Agricultural Sciences, Beijing 100193, China; yueqinqueen@126.com (Y.W.); hekanglai@caas.cn (K.H.); 4 Institute for Multidisciplinary Applied Biology, Universidad Pública de Navarra, Campus Arrosadía, 31192 Mutilva, Navarra, Spain; javier.caballero@unavarra.es (J.C.); pcm92@unavarra.es (P.C.)

**Keywords:** Bacillus thuringiensis, Spodoptera spp., Helicoverpa armigera, Mamestra brassicae, Anticarsia gemmatalis, Ostrinia furnacalis

## Abstract

The bacterium *Bacillus thuringiensis* produces insecticidal Vip3 proteins during the vegetative growth phase with activity against several lepidopteran pests. To date, three different Vip3 protein families have been identified based on sequence identity: Vip3A, Vip3B, and Vip3C. In this study, we report the construction of chimeras by exchanging domains between Vip3Aa and Vip3Ca, two proteins with marked specificity differences against lepidopteran pests. We found that some domain combinations made proteins insoluble or prone to degradation by trypsin as most abundant insect gut protease. The soluble and trypsin-stable chimeras, along with the parental proteins Vip3Aa and Vip3Ca, were tested against lepidopteran pests from different continents: *Spodoptera exigua*, *Spodoptera littoralis*, *Spodoptera frugiperda, Helicoverpa armigera*, *Mamestra brassicae*, *Anticarsia gemmatalis*, and *Ostrinia furnacalis*. The exchange of the Nt domain (188 N-terminal amino acids) had little effect on the stability and toxicity (equal or slightly lower) of the resulting chimeric protein against all insects except for *S. frugiperda*, for which the chimera with the Nt domain from Vip3Aa and the rest of the protein from Vip3Ca showed a significant increase in toxicity compared to the parental Vip3Ca. Chimeras with the C-terminal domain from Vip3Aa (from amino acid 510 of Vip3Aa to the Ct) with the central domain of Vip3Ca (amino acids 189–509 based on the Vip3Aa sequence) made proteins that could not be solubilized. Finally, the chimera including the Ct domain of Vip3Ca and the Nt and central domain from Vip3Aa was unstable. Importantly, an insect species tolerant to Vip3Aa but susceptible to Vip3Ca, such as *Ostrinia furnacalis*, was also susceptible to chimeras maintaining the Ct domain from Vip3Ca, in agreement with the hypothesis that the Ct region of the protein is the one conferring specificity to Vip3 proteins.

## 1. Introduction

*Bacillus thuringiensis* (Bt) is an aerobic, spore-forming, Gram-positive, and entomopathogenic bacterium belonging to the *Bacillus cereus* group. The Bt bacterium produces a wide variety of insecticidal proteins [1] along with other virulence factors contributing to its pathogenicity [2]. Two major categories of insecticidal proteins produced by Bt are δ-endotoxins (Cry and Cyt toxins) that form crystals within the sporangium in the sporulation phase, and vegetative insecticidal proteins (Vip), which are secreted into the growth medium during vegetative growth [1,3,4]. The Vip proteins are classified into four groups (Vip1, Vip2, Vip3, and Vip4) based on their protein sequence similarity [4,5]. The Vip1 and Vip2 proteins act as binary toxins against coleopteran pests [1,4], while for the Vip4 protein no insecticidal activity has been reported yet.

The Vip3 proteins, mainly those of the Vip3A family, are active against a wide range of lepidopteran pests [1,4]. These proteins do not share structural homology with the Cry proteins, but the toxic action follows the same sequence of events: ingestion, activation by midgut proteases, binding to specific receptors in the midgut epithelium, and pore formation [1,4]. Recent studies indicate that Vip3 proteins (either as protoxins or in the activated form of toxin) spontaneously form tetramers in solution [6,7,8,9,10]. In addition, when the Vip3 proteins are activated by proteases, the oligomer remains stable and the cleaved Nt fragment (19 kDa) remains associated to the main fragment (65 kDa) of the protein [6,7,8,9,10]. In agreement with their diverse structure, Vip3 proteins do not share receptors with Cry proteins [11,12,13,14,15], but share receptors with other Vip3 proteins, either from the same (Vip3Aa, Vip3Af, Vip3Ae, and Vip3Ad) or different (Vip3Ca) protein families [14,16].

Five domains have been proposed for the structure of Vip3A proteins from in silico modelling [17,18]. Based on structural features and stability to trypsin, Quan and Ferré [19] identified five domains from Vip3Af: Domain I encompassing amino acids (aa) 12-198, domain II aa 199-313, domain III aa 314–526, domain IV aa 527–668, and domain V aa 669–788. As far as the structural role of the proposed Vip3Af domains, Quan and Ferré [19] found that domains I–III were required to form the tetrameric structure, the role for domain IV was unclear, and domain V was not necessary for oligomerization. Wang et al. [20] generated a disabled Vip3A protein with two site-engineered mutations (S175C and L177C) in domain I, which was not toxic but retained the ability to compete for the wild type binding sites. Taken together, these results suggest that domain I may be involved in post-binding events, such as membrane insertion, and domain V in binding recognition and specificity.

**Figure 1:**
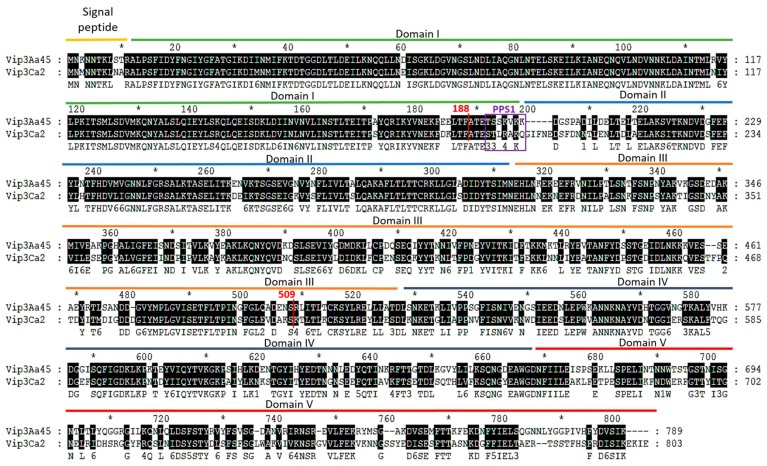
Protein sequence alignment of Vip3Aa45 and Vip3Ca2. Black background shading is used to highlight the conserved amino acid between proteins. The proposed structural domains (based on the Vip3Af proteolysis mutants) are indicated with colored lines above the sequences [19]. The purple box indicates the position of the cleavage site (PPS1), while the red vertical lines show the sites chosen to generate the chimeric proteins.

In this work, we capitalized on the high sequence similarity among Vip3 proteins to test, by domain shuffling, the compatibility of the proposed Vip3Af domains in protein stability and toxicity using representatives from two different Vip3 protein families (Vip3Aa45 and Vip3Ca2). Six chimeric Vip3 proteins (Vip3_ch1, Vip3_ch2, Vip3_ch3, Vip3_ch4, Vip3_ch5, and Vip3_ch6) were designed, where the amino acids (aa) phenylalanine and serine at positions 188 and 509 were chosen as the sites to generate the chimeric Vip3 proteins (Figure 1).

Sequence exchange at these sites coincided approximately with domains I, II+III, and IV+V in the proposed Vip3Af domain model. For the sake of simplicity, we named these domains as the Nt domain (domain I), the central domain (domains II+III), and the Ct domain (domains IV+V), respectively (Figure 1). The objectives of the current research were to determine which main regions of the Vip3 proteins are exchangeable while maintaining the stability and toxicity of the proteins, with the aim to evaluate if any of the new chimeric proteins might confer an increase in the toxicity compared to the parental proteins.

## 2. Results

### 2.1. Sequence Analysis of the Vip3Aa and Vip3Ca Proteins and Determination of the Vip3 Protein Fragment Combinations that Generate Stable Chimeric Proteins

**Figure 2:**
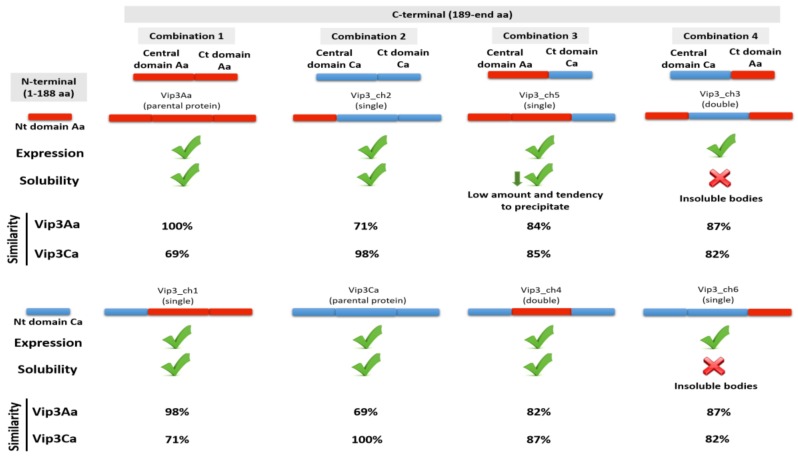
Summary of the combinations of the different Vip3 protein domains expressed in the heterologous *Escherichia coli* expression system. The “single” chimeric Vip3 proteins (Vip3_ch1, Vip3_ch2, Vip3_ch5, and Vip3_ch6) were obtained from the Vip3Aa and Vip3Ca as a template, while the “double” chimeric Vip3 proteins (Vip3_ch3 and Vip3_ch4) were amplified from the Vip3_ch5 and Vip3_ch6. The percentage of similarity of the different proteins vs. the parental proteins, Vip3Aa and Vip3Ca, was calculated with the NCBI Blast align tool.

The amino acid sequence alignment of the two Vip3 proteins indicate that most of the differences are located in their Ct domain (Figure 1). Chimeric proteins were constructed by exchanging the Nt domain (aa 1–188), the central domain (aa 189–509), and the Ct domain (aa 510–788), using as a reference the Vip3A sequence (Figure 1). The Nt domain is highly conserved, with only eight residue differences between the two proteins. The main difference in the protein sequence of the central domain between Vip3Ca and Vip3Aa was two insertions (in Vip3Ca), one located immediately after the main proteolytic processing site (PPS1) (^188^GIFNE), and the other at aa position 464 (^464^TF) [21]. To determine the combinations of the different Vip3 protein domains that generated soluble chimeric Vip3 proteins, all the possible combinations were expressed in *Escherichia coli* (Figure 2). The results indicated that the six chimeric proteins could be expressed, but only the Vip3_ch1, Vip3_ch2, Vip3_ch4, and Vip3_ch5 proteins could be solubilized from the respective inclusion bodies (Figure 2). The exchange of Nt domain did not affect the solubility of the generated chimeric proteins (Vip3_ch1 and Vip3_ch2) (Figure 2). However, the exchange of the Ct domain had, in most cases, a negative effect on the solubility of the chimeric protein. The Ct domain from Vip3Aa combined with the central domain from Vip3Ca produced insoluble proteins (Vip3_ch3 and Vip3_ch6), whereas the reciprocal combination produced a little soluble protein, with tendency to precipitate (Vip3_ch5), and a soluble one (Vip3_ch4) (Figure 2).

### 2.2. Proteolytic and Thermal Stability of The Parental and Chimeric Proteins

**Figure 3:**
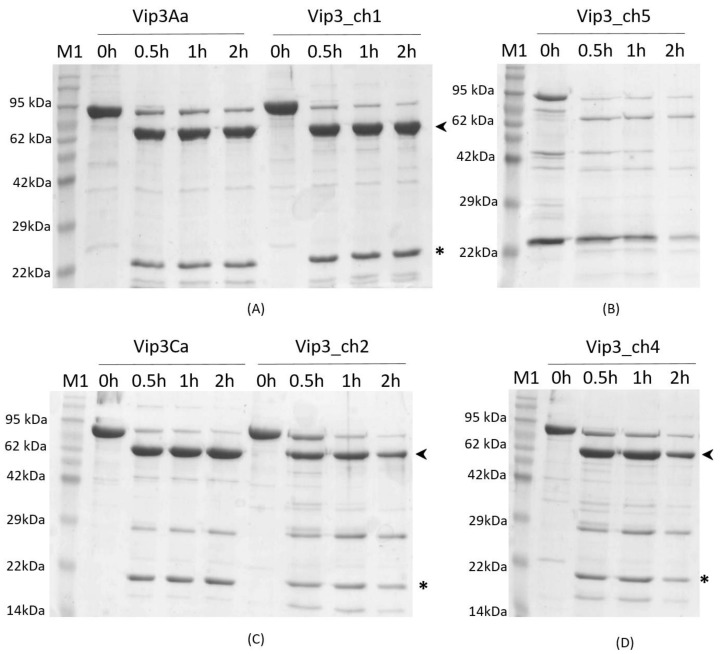
Time course of trypsin activation of Vip3 parental and chimeric protoxins. The reaction was carried out using 1% trypsin (w:w) at 37 °C for increasing incubation periods. (**A**) Vip3Aa protein and Vip3_ch1, (**B**) Vip3_ch5. (**C**) Vip3Ca and Vip3_ch2; (**D**) Vip3_ch4. The arrowheads indicate the protein bands corresponding to the 62–67 kDa fragment, while the asterisks indicate the protein bands corresponding to the 19–22 kDa fragment. M1: Molecular Mass Marker.

To determine whether the chimeric proteins were stable to the activation by proteases, the proteins were digested with 1% trypsin (w:w). The results showed that Vip3Aa, Vip3Ca, and the chimeric proteins Vip3_ch1, Vip3_ch2, and Vip3_ch4 were processed into the two expected protein fragments of 65-67 kDa and 19-22 kDa (Figure 3). However, the proteolytic pattern of the Vip3_ch5 chimera differed from the rest of the Vip3 proteins; this phenomenon could be due to (i) instability to proteases, (ii) instability of the Vip3_ch5 protein in solution, or (iii) problems in the production and purification of the protein. (Figure 3B). Thermal stability of the more soluble and highly purified Vip3 proteins (Vip3Aa, Vip3Ca, Vip3_ch1, Vip3_ch2, and Vip3_ch4) resistant to the trypsin treatment was tested by the thermofluor method (Figure S1). The parental protein Vip3Aa showed two thermal transitions (melting temperature, Tm, of Vip3A-Peak (1): 59.4 ± 0.4 and Tm of Vip3A-Peak (2): 75.5 ± 0.0), while Vip3Ca only showed one thermal transition (Tm of Vip3C-Peak (2): 73.7 ± 0.0) (Figure S1). The chimeric proteins also showed two thermal transitions, but with the first negative peak less pronounced than in the parental Vip3Aa, indicating that the first denaturation involved a lesser part of the protein (Figure S1).

### 2.3. Insecticidal Activity of the Parental and Chimeric Vip3 Proteins

The insecticidal activity of the soluble chimeric proteins (Vip3_ch1, Vip3_ch2, Vip3_ch4, and Vip3_ch5) was compared to that of the parental proteins by testing eight insect species with different susceptibilities to Vip3Aa and Vip3Ca (Table 1). The Vip3Aa protein was toxic for all the insect species tested except for *Ostrinia furnacalis* (for this insect species the Vip3Aa protein is only toxic at very high concentration). The Vip3Ca protein showed high toxicity to *O. furnacalis* and moderate toxicity to *A. gemmatalis*; for the other insect species tested, this protein was slightly active at very high concentrations (Table 1).

Regarding the chimeric proteins, the exchange of the Nt domain in Vip3Aa (Vip3_ch1 chimera) decreased the insecticidal activity (compared to Vip3Aa) against all the insect species tested (detected when testing at lower concentrations 0.5 and 0.3 ug/cm^2^, 0.3 ug/mL and 5 ug/g) except for *S. exigua.* In the case of *A. gemmatalis* and *O. furnacalis*, this chimera completely lost toxicity (Table 1). In the case of the Vip3Ca protein, the exchange of the Nt domain (Vip3_ch2 chimera) led to different outcomes depending on the insect species considered. Insecticidal activity did not significantly differ from that of Vip3Ca in *S. exigua*, *S. littoralis*, *H. armigera.* The insecticidal activity of the Vip3_ch2 chimera decreased in *A. gemmatalis*, *M. brassicae*, and *O. furnacalis.* Most interestingly, in the case of *S. frugiperda* the Vip3_ch2 chimera showed a strong gain of function compared to the Vip3Ca parental protein with mortality values similar to the ones of the most active parental protein, Vip3Aa (Table 1). Chimera Vip3_ch4 (with the central domain from Vip3Aa and the flanking ones from Vip3Ca) was nontoxic for any of the insect species tested, except for *O. furnacalis* (Table 1). In the case of Vip3_ch5, the chimeric protein did not cause any damage to any of the insects tested, most likely due to the instability of this protein or problems in the production and purification.

The toxicity of the three proteins active against *O. furnacalis* was confirmed by determining the LC_50_ values (Table 2 and Table S1). The results indicated that, though similarly toxic, Vip3Ca was the most toxic protein (LC_50_ = 1.2 µg/g) followed by Vip3_ch2 (LC_50_ = 2.3 µg/g) and Vip3_ch4 (LC_50_ = 3.9 µg/g). In the case of the chimera with gain of function for *S. frugiperda*, the LC_50_ value was determined and compared to the most toxic parental protein, Vip3Aa. The results indicated that the toxicity of Vip3_ch2 (LC_50_ = 133 ng/cm^2^) did not significantly differ from that of Vip3Aa (LC_50_ = 162 ng/cm^2^), but was significantly increased compared to the Vip3Ca protein (LC_50_ > 7000 ng/cm^2^) (Table 1, Table 2 and Table S1).

## 3. Discussion

Insecticidal proteins in the Vip3A family have been incorporated in commercial transgenic crop varieties [23] due their potent and broad spectrum activity against lepidopteran pests [4]. In contrast, members of the Vip3B and Vip3C protein families have a narrow insecticidal spectrum and a moderate activity [8,21,24,25,26,27,28]. In the case of Vip3Ca, among the 25 species of insects tested [21,25,26,27,28], only for *O. furnacalis* and *Mythimna separata* its toxicity was comparable to the most toxic Cry or Vip3 proteins (Cry1Ab for *O. furncacalis* or Vip3Aa for *M. separata*) [26,27]. The present work focused on testing the compatibility of domains exchanged between a member of the Vip3A family and one of the Vip3C family, with the possibility of generating novel proteins with increased insecticidal activity.

The results show that the exchange of the Nt domain does not affect the solubility and trypsin stability of the resulting chimeric Vip3 proteins (Vip3_ch1 and Vip3_ch2) (Figure 2). Similar results were obtained in another study testing the exchange of the Nt domain between Vip3Ab and Vip3Bc [8]. This is not a surprising result since the Nt domain is extremely conserved among Vip3 proteins, suggesting a structural role or a possible role in post binding events, such as pore formation. Wang et al. [20] generated a Vip3A protein mutated at the Nt domain (S175C and L177C) which was able to compete with binding of the wild type protein but not cause mortality, thus supporting the previous hypothesis. With regards to the Ct domain, of the four chimeras produced only one was soluble and stable to treatment with trypsin (Figure 2). These results suggest that the interaction of the Ct domain with the other domains in the 3D structure of Vip3 is more specific and critical to the physicochemical properties of the molecule. Furthermore, results from thermofluor assays showed that the chimeric proteins had a thermal stability intermediate between that of the two parental proteins and that the Tm values and the presence/intensity of one or two thermal transitions depended on the interaction between the respective Vip3 domains (Figure S1). Specifically, the denaturation curve profile for the Vip3Aa, Vip3_ch1, Vip3_ch2, and Vip3_ch4 proteins indicates that these proteins have two motifs with different thermal stability, while the Vip3Ca protein would be more stable because of its single denaturation curve. Further understanding of the structure in this family of proteins would shed light on this aspect.

The results from the insecticidal spectrum of the chimeric proteins indicated that, in general (and considering that we only tested one species in the Crambidae family), the activity follows taxonomical relationships at the family level. Thus, species in Noctuidae presented a closer susceptibility profile to both parental and chimeric proteins when compared to the tested species in Crambidae (Table 1). This observation is in agreement with the results of Zack et al. [8], where the Noctuid insects (*Helicoverpa zea*, *S. frugiperda*, and *Pseudoplusia includens*) showed more similar susceptibility profiles for the parental (Vip3Ab and Vip3Bc) proteins and their chimeric proteins (generated by exchange of the Nt domain), compared to the Crambidae insects (*Ostrinia nubilalis*). That study also showed that the chimeras were less toxic than the parental proteins to *H. zea*, *S. frugiperda*, *O. nubilalis*, and *P. includens* [8]. Similarly, our results with the chimeras indicate that, with the exception of Vip3_ch2, the chimera proteins are similarly or less toxic than the parental proteins (Table 1). The Vip3_ch2 chimera, a Vip3C protein with the Nt domain from Vip3Aa, displayed gain of function only with *S. frugiperda* but not with other closely related species of the same genus (Table 1). A similar result was recently reported in which a “modified Vip3C protein” (i.e., ARP150v2, 98% similarity to the Vip3_ch2) had higher toxicity against *S. frugiperda* than the parental Vip3Ca protein [29]. Sequence analysis indicates that ARP150v2 is a chimera in which the Nt domain of Vip3Ca has been replaced by that of Vip3Af1. We do not have a clear explanation for this unique increase in toxicity, but due to the specificity of the phenomenon, the reason has to be more efficient interaction with the receptors and/or facilitated post-binding events, such as membrane insertion or pore formation. Further research testing the mode of action of this family of proteins should clarify this particular phenomenon.

## 4. Conclusions

In summary, we present evidence for the relative importance of different Vip3 protein domains in stability and toxicity, and an example of how the design of chimeric Vip3 proteins may lead to novel proteins with improved and expanded insecticidal activity. Specifically, the Vip3_ch2 protein, a Vip3C protein with the Nt domain from Vip3Aa, showed a gain of function for *S. frugiperda*. In addition, the Vip3_ch4 protein showed that for the toxicity of the Vip3C protein in *O. furnacalis*, the specificity is provided by the Ct domain.

## 5. Materials and Methods 

### 5.1. Design and Construction of Chimeric Vip3 Proteins

An overlap PCR method was used to generate the chimeric proteins from the parental Vip3Aa45 (JF710269.1) and Vip3Ca2 (JF916462.1) proteins [21,30]. To construct the Vip3A and Vip3C chimeric proteins, amino acids (aa) stretches at positions 188 (^188^FATET) and 509 (^509^SRLIT) of the Vip3Aa protein were used to define the protein fragments to exchange: fragment I (aa 0 - 188), fragment II (aa 189 - 508), fragment III (aa 509 - 788) (Figure 1). Six chimeric proteins were generated and classified as “single” (Vip3_ch1, 2, 5, and 6) or “double” (Vip3_ch3 and 4), depending on whether they were amplified from the parental or the Vip3_ch5 and 6 proteins, respectively (Table S2).

To generate the chimeric genes, first a PCR was performed to amplify the necessary fragments separately with the annealing primers (Tables S2 and S3). The PCR reaction contained, in a final volume of 50 µL, 50 ng of the DNA template, 0.25 U of Kapa Hifi DNA polymerase, 5 µL of five-fold reaction buffer, 0.6 mM of each dNTPs, and 0.3 µM of the respective primers. PCR amplifications were carried out as follows: 5 min denaturation at 95 °C, 35 cycles of amplification ((20 s of denaturation at 98 °C, 15 s of annealing at 60 °C, and 30 s of extension at 72 °C), and an extra extension step of 5 min at 72°C). The amplicons were purified form the agarose gel and a second PCR (overlap step + “amplification step”) was performed with the respective DNA fragments (Table S2). First, the “overlap step” was conducted in a final volume of 50 µL with 100 ng (total amount) of the respective DNA fragments (Table S2) in an equimolecular ratio, 0.25 U of Kapa Hifi DNA polymerase, 5 µL of five-fold reaction buffer, 0.6 mM of each dNTPs. PCR amplifications were carried out as follows: 5 min denaturation at 95 °C, 15 cycles of amplification ((20 s of denaturation at 98 °C, 30 s (Vip3_ch1, 2, 4, 5, and 6)/1 min (Vip3_ch3) of annealing at 55 °C (Vip3_ch1, 2, 4, 5, and 6) or 50 °C (Vip3_ch3), 2 min of extension at 72 °C) and an extra extension step of 5 min at 72 °C). Second, the “amplification step” was performed with the respective end primers (Table S2), adding 0.3 µM of each to the PCR reactions. The PCR reactions were carried out in the conditions described for the “overlap step”. In addition, for the Vip3_ch3 protein a nested-PCR with the DNA amplified in the second PCR was carried out (PCR reaction: final volume 50 µL, 7 ng of the Vip3 chimera 3, 0.25 U of Kapa Hifi DNA polymerase, 5 µL of five-fold reaction buffer, 0.6 mM of each dNTPs, and 0.3 µM of the respective primers (Table S2). Conditions for this nested-PCR amplification were 5 min denaturation at 95 °C, 35 cycles of amplification (20 s of denaturation at 98 °C, 60 s of annealing at 50 °C, and 2 min of extension at 72 °C), and an extra extension step of 5 min at 72 °C). Amplicons were purified from an agarose gel, ligated into the pGEM^®^-T Easy plasmid or pCR^®^2.1-TOPO^®^, cloned in *E. coli* DH10β, and sequenced with the sequencing primers (Table S3).

For expression, the full length genes were amplified from the pGEM^®^-T Easy or pCR^®^2.1-TOPO^®^ with the end primers (Table S3). The PCR reactions contained, in a final volume of 50 µL, 50 ng of the respective Vip3 chimeric genes, 0.25 U of Kapa Hifi DNA polymerase, 5 µL of five-fold reaction buffer, 0.6 mM of each dNTPs, and 0.3 µM of the respective end primers (Table S2). Conditions for PCR amplifications were as follows: 5 min denaturation at 95 °C, 35 cycles of amplification ((20 s of denaturation at 98°C, 60 s of annealing at 55 °C (Vip3_ch1, 2, 4, 5, and 6) 50 °C (Vip3_ch3), 2 min of extension at 72 °C), and an extra extension step of 5 min at 72 °C). Amplicons were purified and together with the expression vector (pET30a (+)) were digested with BamHI and NotI for 2 h at 37 °C. The pET30a (+) plasmid was dephosphorylated for 2 h at 37 °C with alkaline phosphatase. The linearized/dephosphorylated pET30a (+) and the digested chimeric genes were purified prior to ligation using T4 DNA Ligase overnight at 4 °C. Ligation reactions were transformed in *E. coli* BL21 (D3) and transformants confirmed by sequencing with the sequencing primers (Table S3).

### 5.2. Expression and Purification of Vip3Aa, Vip3Ca, and Chimeric Proteins

#### 5.2.1. Expression of the Parental and Chimeric Vip3 Proteins

The Vip3Ca protein was expressed following the conditions described by Gomis-Cebolla et al. (2017) [16]. For expression of Vip3Aa and the chimeric proteins, a single colony was inoculated in 7 mL of LB-K medium (LB medium containing 50 μg/mL kanamycin) and grown overnight at 37 °C and 180 rpm. A 1/100 dilution of the culture in 700 mL LB-K medium was further incubated at 37 °C and 180 rpm. When the OD was 0.7–0.8, 1 mM IPTG (final concentration) was added for induction. Induced cultures were grown overnight at 37 °C and 180 rpm, and the cells were collected by centrifugation at 8800× *g* for 30 min at 4 °C. Cell pellets for the Vip3Aa and Vip3Ca proteins were lysed by chemical lysis. Briefly, three milliliters of lysis buffer-I (50 mM sodium phosphate buffer, 0.5 M NaCl, pH 8.0, containing 3 mg/mL lysozyme, 10 µg/mL DNase, 10 mM DTT, and 100 μM PMSF) per gram of pellet were added to the samples. The pellets were resuspended with an Ultra Turrax T25 digital homogenizer (IKA, Janke & Kunkel-Str. 10 Staufen, DE) at 16,000× *g* and incubated at 37 °C for 60 min with strong shaking (200 rpm). Then, the lysate was sonicated on ice applying five cycles (1 min pulse at 70 W, 10 s off, 1 min pulse at 70 W). Insoluble materials were separated by centrifugation at 31,000× *g* for 15 min and 4 °C. The soluble cellular fractions were filtered through sterile 0.45 μm cellulose acetate filters. In the case of chimeric proteins, three milliliters of lysis buffer-II (50 mM sodium phosphate buffer, 0.5 M NaCl, pH 8.0, containing 3 mg/mL lysozyme, 10 mM DTT and 100 μM PMSF) per gram were added to the pellets and the samples were resuspended as described above, and then incubated at 37 °C for 60 min with strong shaking (200 rpm). After incubation, 8 mg of deoxycholic acid sodium salt per gram of pellet was added and incubated 30 min at 37 °C with gentle shaking (100 rpm), after which 40 µg/mL of DNase was added to eliminate the viscosity of the lysates and further incubated for 30 min at 37 °C with gentle shaking. The lysates of the chimeric proteins were then sonicated on ice applying five cycles (1 min pulse at 70 W, 10 s off, 1 min pulse at 70 W), centrifuged at 31,000× *g* for 15 min at 4 °C and the soluble cellular fraction was filtered through sterile 0.45 μm cellulose acetate filters. In the case of the Vip3 chimeras 3 and 6, they formed inclusion bodies that were not possible to dissolve in the conditions used in the present study (Figure S2).

#### 5.2.2. Purification of Vip3Aa, Vip3Ca, and Chimeric Vip3 Proteins by Isoelectric Point Precipitation

**Figure 4:**
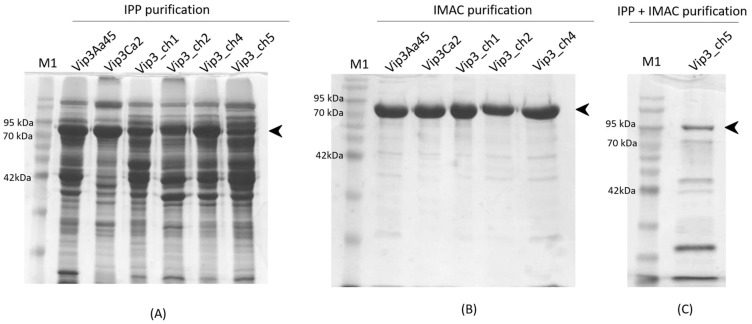
SDS-PAGE analysis of the purified parental (Vip3Aa and Vip3Ca) and chimeric (Vip3_ch1, Vip3_ch2, Vip3_ch4, and Vip3_ch5) proteins. (**A**) Parental proteins and chimeric Vip3 proteins (5 µg) purified by isoelectric point precipitation (IPP). (**B**) Parental proteins and chimeric Vip3 proteins (5 µg) purified by ion metal affinity chromatography (IMAC) on a Hi-Trap chelating HP column (GE Healthcare). (**C**) Vip3_ch5 protein (2 µg) purified by IPP and IMAC on a Hi-Trap chelating HP column (GE Healthcare). The arrowheads indicate the protein band corresponding to the chimeric Vip3 proteins. M1: Molecular Mass Marker.

For the insect toxicity assays, two independent batches of the Vip3Aa, Vip3Ca and the chimeric proteins (Vip3_ch1, Vip3_ch2, Vip3_ch4, and Vip3_ch5), were purified by isoelectric point precipitation (IPP) in three steps (Figure 4A) [31]. First, the soluble cellular fractions of the Vip3 proteins were diluted three-fold with 50 mM sodium phosphate buffer pH 8.0, dialyzed overnight against the dialysis buffer (20 mM sodium phosphate buffer, 150 mM NaCl, pH 8), centrifuged at 14,000× *g* for 15 min at 4 °C, and then filtered through 0.45 μm cellulose acetate filters. Second, the pH of the respective lysates was lowered with acetic acid to pH 5.5 for Vip3Aa, pH 5.9 for Vip3Ca, pH 5.0 for Vip3_ch1, pH 5.5 for the Vip3_ch2, pH 5.2 for the Vip3_ch4, pH 5.2 for the Vip3_ch5. The Vip3 proteins were recovered by centrifugation (14,000× *g* for 15 min at 4 °C). Third, the pellets were resuspended in storage buffer (20 mM Tris buffer, 150 mM NaCl pH 8.6) for 1 h with shaking at 4 °C, and then centrifuged (14,000× *g* for 15 min at 4 °C) and filtered through 0.45 μm cellulose acetate filters. The Vip3 proteins were quantified by densitometry and the ratio of Vip3 protein/total protein (w:w) was calculated. The samples were stored at −80 °C and lyophilized prior to their use or shipping at room temperature to other laboratories.

#### 5.2.3. Purification of Vip3Aa, Vip3Ca, and Chimeric Vip3 Proteins by Ion Metal Affinity Chromatography

For the proteolysis and thermal shift assays, the parental proteins (Vip3Aa and Vip3Ca) and the chimeric Vip3 proteins (Vip3_ch1, Vip3_ch2, Vip3_ch4, and Vip3_ch5) were purified by ion metal affinity chromatography (IMAC) on a His-Trap FF crude lysate column (GE Healthcare) (Figure 4B). The soluble cellular fractions of the Vip3 proteins (Vip3Aa, Vip3Ca, Vip3_ch1, 2, 4, and 5) were diluted three-fold with 50 mM sodium phosphate buffer pH 8.0, dialyzed against the dialysis buffer to eliminate the presence of DTT and deoxycholic acid. Samples were dialyzed for 10-16 h at 4 °C, and the dialysis buffer exchanged twice. The unclarified lysates were filtered through 0.45 μm cellulose acetate filters to discard protein aggregates. The soluble Vip3 protein fractions were loaded into a His-Trap FF crude lysate column equilibrated in binding buffer (20 mM phosphate buffer, 150 mM NaCl, 10 mM imidazole, pH 8). After washing the column with binding buffer to eliminate unbound molecules, Vip3 proteins were eluted using elution buffer (20 mM phosphate buffer, 150 mM NaCl, 150 mM imidazole, pH 8) into 2 mL tubes containing 0.1 mM EDTA (pH 8.0). 

Since the Vip3_ch5 was expressed in low quantities (data not shown), first the protein was partially purified by IPP as described above, and then filtered through 0.45 μm cellulose acetate filter, prior to loading into the His-Trap FF crude lysate column (Figure 4C).

To avoid protein precipitation, buffer exchange was performed against storage buffer (20 mM Tris 500 mM NaCl, pH 8.6) by dialysis overnight at 4 °C. The concentration and quality of the purified proteins were estimated with the Bradford assay [32] using BSA as standard and by SDS-PAGE, respectively. The Vip3 proteins were snap frozen in liquid nitrogen and stored at −80 °C until used.

### 5.3. Thermal and Protease Stability of the Parental and the Chimeric Vip3 Proteins 

The parental (5 µg) and chimeric (5 µg of Vip3_ch1, Vip3_ch2, Vip3_ch4, and 2 µg of Vip3_ch5) proteins were subjected to proteolysis with 1% (w/w) bovine trypsin (SIGMA T8003, Sigma-Aldrich, Madrid, Spain) for different time intervals (0, 0.5, 1, and 2 h) at 37 °C. The proteolytic reactions were stopped with 1 mM of AEBSF protease inhibitor for 10 min at room temperature, and then the samples were resolved by SDS-12%PAGE and stained with Coomassie brilliant blue R-250 (Sigma 1125530025, Sigma-Aldrich, Madrid, Spain). The size of the protoxin and trypsin-activated fragments were analyzed using the TotalLab 1D v 13.01 software.

The Tm of the parental proteins and the chimeric proteins resistant to trypsin treatment were determined using the environmentally sensitive extrinsic dye SYPRO-Orange [33]. The thermal shift reactions were prepared at room temperature (RT) and contained, in a final volume of 180 µL, 1 µM of the respective Vip3 proteins (filtered through 0.45 µm cellulose acetate filter), 15× of SPYRO-Orange (diluted in storage buffer-I) and storage buffer-I up to 180 µL. Eight replicates (20 µL) of the parental and chimeric proteins plus a negative control (15× of SPYRO-Orange and storage buffer-I) were incubated for 5 min at RT and centrifuged at 141× *g* for 1 min prior to analysis with the StepOnePlus™ Real-Time PCR System (Thermo Fisher Scientific, Waltham, MA, USA). Thermal shift assays program was carried out as follows: Reporter: ROX; Passive Reference: None; Run Method: Mode Continuous, Program 2 min at 25 °C, Temperature Ramp 4% (4 °C/min), 2 min at 99 °C. The data were exported to an Excel file to determine the Tm of the respective Vip3 proteins by plotting the negative of the first derivative of the fluorescence as a function of temperature-dFv/dT, where Fv and T at (t+1)-t represent the increment of fluorescence and temperature between each measurement.

-dFv/dT = (Fv(t + 1) − Fv(t))/(T(t + 1) − T(t))(1)

The Tm values of the respective Vip3 proteins were compared with one-way ANOVA analysis and datasets statistically significant (α < 0.05), were analyzed by the multiple comparison Tukey post hoc test (α < 0.05).

### 5.4. Insect Colonies and Toxicity Assays

Insects were reared and bioassays performed at the insectaries of the University of Valencia (for *S. exigua* and *S. littoralis*, Spain), Public University of Navarra (for *H. armigera, M. brassicae*, Spain), University of Tennessee (for *S. frugiperda* and *A. gemmatalis*, Knoxville, TN, USA), and Chinese Academy of Agricultural Sciences (for *O. furnacalis*, Haidan district, Beijing, China) at 25 °C, 70% RH, 16:8 L/D photoperiod (*S. exigua*, *S. frugiperda*, *S. littoralis*, *M. brassicae*, *H. armigera*, and *A. gemmatalis*) and 27 °C, 80% RH, 16:8 h L/D photoperiod (*O. furnacalis*), respectively. The insect colonies of *S. exigua, S. littoralis*, *H. armigera*, *M. brassicae*, and *O. furnacalis* had been reared for several generations in laboratory conditions without exposure to any insecticide. In the case of *S. frugiperda* and *A. gemmatalis* the insects were purchased from Benzon Research Inc. (Carlisle, PA, USA). The laboratory insect colonies of *S. exigua*, *S. littoralis*, *M. brassicae*, and *H. armigera* were reared in a growth wheat germ-based semi-synthetic diet [34], while *O. furnacalis* had been reared using standard rearing techniques without exposure to any insecticide [35]. In the case of *S. frugiperda* and *A. gemmatalis*, they were reared with meridic diet (#F9772, Frontier Agricultural Sciences, Newark, DE, USA). The same diets and rearing conditions were used in the bioassays with the parental proteins and chimeric Vip3 proteins.

Different methodologies were used in the bioassays depending on the insect species tested. For *S. exigua*, *S. littoralis*, *S. frugiperda*, *H. armigera*, and *A. gemmatalis*, bioassays were performed on neonates using surface contamination. Briefly, two pairs of different concentrations were dispensed on the diet surface. Prior to the sample application, the surface of the diet was sterilized under UV light for 10 min. A volume of 50–75 µL of each concentration was applied on the surface of solidified diet (2 cm^2^ multiwell plates, Bio-Cv-16, C-D International) and let dry in a flow hood. Once dried, one larva was transferred to each well using a brush. In the case of *O. furnacalis*, bioassays were performed on neonates using diet incorporation assays [36], while for *M. brassicae* bioassays were performed on L2 instar larvae using a droplet feeding method [37]. To determine the effect of the domain exchange on toxicity, bioassays with Vip3 proteins were performed with two different concentrations (chosen as to give a discriminant mortality, range of mortality for each insect species between 1% and 99%) in at least two different insect generations (Table 1). Thirty-two neonates were used for each protein concentration for *S. exigua*, *S. littoralis*, *S. frugiperda*, and *A. gemmatalis*; 28 in the case of *H. armigera* and *M. brassicae*; for *O. furnacalis* 42 neonates were tested. Mortality (number of dead larvae) was scored after 7 days for *S. exigua*, *S. littoralis*, *S. frugiperda*, *H. armigera*, *M. brassicae*, and *O. furnacalis*; while for *A. gemmatalis* mortality was scored after 5 days. Only data from bioassays with <10% control mortality were considered.

Determination of the LC_50_ (concentration of protein killing 50% of tested individuals) for the toxic parental and chimeric proteins was done for *O. furnacalis* (concentration range 0.04–50 µg/g) and *S. frugiperda* (concentration range 0.01–3 µg/cm^2^). For *S. frugiperda* a set of 16–32 neonates per concentration (7–8 concentrations of the respective Vip3 proteins) were tested under the same conditions as described above for bioassays, and bioassays replicated twice. The number of dead larvae was recorded after 7 days of exposure. In the case of *O. furnacalis*, neonates were introduced to individual wells of 48-well trays containing 9–11 concentrations of purified toxin, which were tested with a total of 96 larvae per concentration. Trays were incubated as per the rearing conditions above and mortality and survivor weight were recorded after 7 days of exposure. Bioassays were repeated with two insect generations. The storage buffer was used to dilute the parental and chimeric Vip3 proteins and as negative control. Bioassay data were subjected to nonlinear regression using the software GraphPad Prism7 to obtain the LC_50_ of the parental proteins and chimeric Vip3 proteins, which were compared the parental proteins vs. the chimeric proteins with the statistical analysis extra-sum-square *F* test (α 0.05) (Table S1).

## Figures and Tables

**Table 1:** Susceptibility of lepidopteran insect pests to parental (Vip3Aa and Vip3Ca) and chimeric proteins.

Insect Family	Insect Genus	Insect Species (Instar Tested)	Concentration	% of Corrected Mortality (Mean ± SD *) ^ẞ^					
				Vip3Aa	Vip3Aa Chimeras		Vip3Ca	Vip3Ca Chimeras	
					Vip3_ch1	Vip3_ch5		Vip3_ch2	Vip3_ch4
			µg/cm^2^						
Noctuidae	*Spodoptera*	*S. exigua* (neonate)	4.1	85.9 ± 4.6	86.7 ± 3.3	6.5 ± 0.2	-	-	-
			0.5 ^ζ^	70.8 ± 10.4 (a)	68.2 ± 0.5 (a)	6.3 ± 6.3 (b)	-	-	-
			7	-	-	-	51.8 ± 1.4	41.5 ± 10.3	9.4 ± 0.0
			0.7 ^ζ^	-	-	-	29.7 ± 1.6 (c)	32.6 ± 4.5 (c)	3.4 ± 0.3 (d)
							
		*S. littoralis* (neonate)	4.1	98.4 ± 1.6	100.0 ± 1.6	7.8 ± 1.6	-	-	-
			0.5 ^ζ^	98.4 ± 1.6 (b)	70.0 ± 17.0 (c)	3.1 ± 3.1 (d)	-	-	-
			7	-	-	-	32.3 ± 2.1	34.3 ± 12.6	4.6 ± 1.6
			0.7 ^ζ^	-	-	-	6.3 ± 6.3 (e)	11.5 ± 0.8 (e)	4.7 ± 4.7 (f)
							
		*S. frugiperda* (neonate)	4.1	97.0 ± 3.1	98.4 ± 1.6	1.6 ± 1.6	-	-	-
			0.5 ^ζ^	91.0 ± 0.0 (g)	59.4 ± 9.4 (h)	1.6 ± 1.6 (i)	-	-	-
			7	-	-	-	53.0 ± 6.6	98.4 ± 1.6	1.6 ± 1.6
			0.7 ^ζ^	-	-	-	6.2 ± 6.2 (j)	92.2 ± 7.8 (k)	1.6 ± 1.6 (l)
								
	*Helicoverpa*	*H. armigera*^‡^ (neonate)	2.5	100 ± 0.0	66.5 ± 11.5	17.0 ± 4.0	-	-	-
			0.3 ^ζ^	83.5 ± 3.5 (m)	9.0 ± 1.0 (o)	6.5 ± 2.5 (p)	-		-
			4	-	-	-	61.5 ± 9.5	82.0 ± 4.0	2.0 ± 2.0
			0.4 ^ζ^	-	-	-	15.5 ± 5.5 (q)	15.0 ± 2.0 (q)	1.0 ± 1.0 (r)
			µg/cm^2^						
Noctuidae	*Anticarsia*	*A. gemmatalis* (neonate)	4.1	98.4 ± 1.5	6.3 ± 6.3	7.8 ± 4.7	-	-	-
			0.5 ^ζ^	98.4 ± 1.5 (s)	0.0 ± 0.0 (t)	6.3 ± 6.3 (v)	-	-	-
			7	-	-	-	90.0 ± 3.3	54.7 ± 14.1	7.8 ± 7.8
			0.7 ^ζ^	-	-	-	54.7 ± 11.8 (w)	21.9 ± 6.3 (y)	1.6 ± 1.6 (z)
								
			µg/ml						
	*Mamestra*	*M. brassicae*^‡^ (L2)	2.5	79.3 ± 0.9	69.0 ± 6.0	22.0 ± 7.0	-	-	-
			0.3 ^ζ^	56.5 ± 8.5 (aa)	31.0 ± 2.0 (ab)	14.0 ± 1.0 (ac)	-	-	-
			4	-	-	-	65.6 ± 12.3	26.0 ± ND ^δ^	15.5 ± 4.0
			0.4 ^ζ^	-	-	-	30.5 ± 1.5 (ad)	7.0 ± ND ^δ^ (ae)	10.0 ± 1.0 (af)
									
			µg/g						
Crambidae	*Ostrinia*	O. furnacalis * (neonate)	50	55.2 ± 1.0	14.6 ± 4.2	21.8 ± 3.1	96.8 ± 1.0	98.9 ± 1.0	85.4 ± 2.1
			5 ^ζ^	16.6 ± 4.2 (ah)	8.3 ± 0.0 (ai)	16.6 ± 0.0 (ah)	91.6 ± 2.1 (aj)	79.2 ± 2.1 (ak)	65.6 ± 1.0 (al)

* Standard deviation of the mean. ^δ^ ND: not possible to calculate the standard deviation of the mean of the Vip3_ch2 in *M. brassicae* colony because the assay was done with one replicate. ^ẞ^ The percentage of mortality in the different treatments was corrected by subtracting the value of mortality observed for the buffer treatment (negative control). ^‡^ In the case of *H. armigera* and *M. brassicae*, the concentration used in the surface contamination and droplet feeding method assays were adapted from Ruiz de Escudero et al. 2014 [22] (2.5 and 0.3 µg/cm^2^) and Palma et al. 2012 [21] (4 and 0.4 µg/cm^2^). ^ζ^ For each insect species, the mortality values at lowest dose (discriminant concentration used) followed by the same letter were not statistically different based on the based on the overlap of standard deviation of the mean.

**Table 2:** Determination of the lethal concentration (LC_50_) of the parental and selected chimeric Vip3 proteins in *Ostrinia furnacalis* and *Spodoptera frugiperda*.

Insect Species	Toxin	Number of Insects Tested	Slope Factor			Lethal Concentration			Goodness of Fit			
			Slope	SE *	CI_95_ †	LC_50_^ζ^	SE *	CI_95_ †	R^2^	Absolute Sum Squares	Sy.x ^‡^	Df ^¥^
***O. furnacalis***						µg/g						
	Vip3Ca	768	1.4	0.06	1.3–1.6	1.2 (a)	1.0	1.1–1.3	0.99	129	2.7	18
	Vip3_ch2	768	1.0	0.05	0.9–1.1	2.3 (b)	1.0	2.0–2.5	0.99	195	3.3	18
	Vip3_ch4	768	1.2	0.07	1.0–1.3	3.9 (c)	1.0	3.5–4.5	0.98	327	4.3	18
						ng/cm^2^						
*S. frugiperda*	Vip3Aa	512	1.6	0.25	1.1–2.2	162.0 (d)	1.1	130–202	0.95	1616	9.2	19
	Vip3_ch2	336	1.6	0.25	1.1–2.2	133.1 (d)	1.1	107–166	0.97	1086	8.2	16

* Standard error of the slope and lethal dose concentration, respectively † Confidence interval at 95% for the slope and lethal dose concentration, respectively. ^‡^ Quantification of the standard deviation of the residuals (vertical distance of the point from the fit line or curve) expressed as % of mortality. At higher value, the data shows a greater variance and lower goodness of fit (R^2^). ^¥^ Degree of freedom. ^ζ^ For each insect species, the LC values followed by the same letter were not statistically different based on of the statistical analysis extra-sum-square F test analysis (α 0.05) (Table S1).

## Supplementary Materials

The following are available online at https://www.mdpi.com/2072-6651/12/2/99/s1, Figure S1. Thermal shift assays and multiple comparison of the thermal transitions of the parental and chimeric proteins. The dashed vertical lines in the thermal shift assays curves indicate the Tm (measured in °C) of respective thermal transitions. C- indicates the fluorescence intensity due to the SPYRO-Orange 15× in 20 mM Tris 500 mM NaCl pH 8.6. The thick line indicates the comparison of the Tm by one-way ANOVA (α 0.05). The dashed line indicates the multiple comparison analyzed by Tukey’s range test (α 0.05). “****” indicates a p value less than 0.0001 and “ns” indicates a p value greater than 0.05. Figure S2. Expression of the chimeric Vip3 proteins (Vip3_ch3 and Vip3_ch6). (A) SDS-PAGE of different dilutions of the pellet and supernatant of the Vip3_ch3 and Vip3_ch6 proteins. (B) Western blot analysis of different dilutions of the pellet and supernatant of the respective chimeric Vip3 proteins. The dilutions of the lysates were made with 50 mM phosphate buffer, 500 mM NaCl (pH 8.0), while the pellets were dissolved in the same volume of the supernatant and the dilutions were made with 50 mM phosphate buffer, 500 mM NaCl (pH 8.0). The arrowhead indicates the protein band corresponding to the chimeric Vip3 proteins. M1: Molecular Mass Marker “PINK Plus Prestained Protein Ladder” (Genedirex). M2: Molecular Mass Marker “Precision Plus Protein™ Dual Color Standards” (Biorad) developed with “Precision Protein™ Strep Tactin-HRP conjugate. Availability of data and material: Sequences encoding the Vip3 chimeras have been deposited in GenBanK with the following accession numbers: Vip3 chimera 1 (MH363727), Vip3 chimera 2 (MH363728), Vip3 chimera 3 (MH363729), Vip3 chimera 4 (MH363730), Vip3 chimera 5 (MH363731), and Vip3 chimera 6 (MH363732). Table S1. Comparison analyses of the respective dose-response assays (LC50 values) of the parental and chimeric Vip3 proteins in S. frugiperda and O. furnacalis. Table S2. Construction of the chimeric Vip3 proteins from the Vip3Aa and Vip3Ca proteins. Table S3. Primers used in construction and sequencing of the genes encoding the chimeric Vip3 proteins.
