# Supplementary material for: Domain Shuffling between Vip3Aa and Vip3Ca: Chimera Stability and Insecticidal Activity against European, American, African, and Asian Pests
Source: Toxins (Basel). 2020 Feb 4;12(2):99. doi: 10.3390/toxins12020099 (PMC7076965; doi:10.3390/toxins12020099)
Supplement: Supplementary file 1 [file toxins-12-00099-s001.pdf]

Supplementary Materials: Domain Shuffling between Vip3Aa and Vip3Ca: Chimera Stability and Insecticidal Activity against European, American, African, and Asian Pests

Joaquín Gomis-Cebolla, Rafael Ferreira dos Santos, Yueqin Wang, Javier Caballero, Primitivo Caballero, Kanglai He, Juan Luis Jurat-Fuentes and Juan Ferré

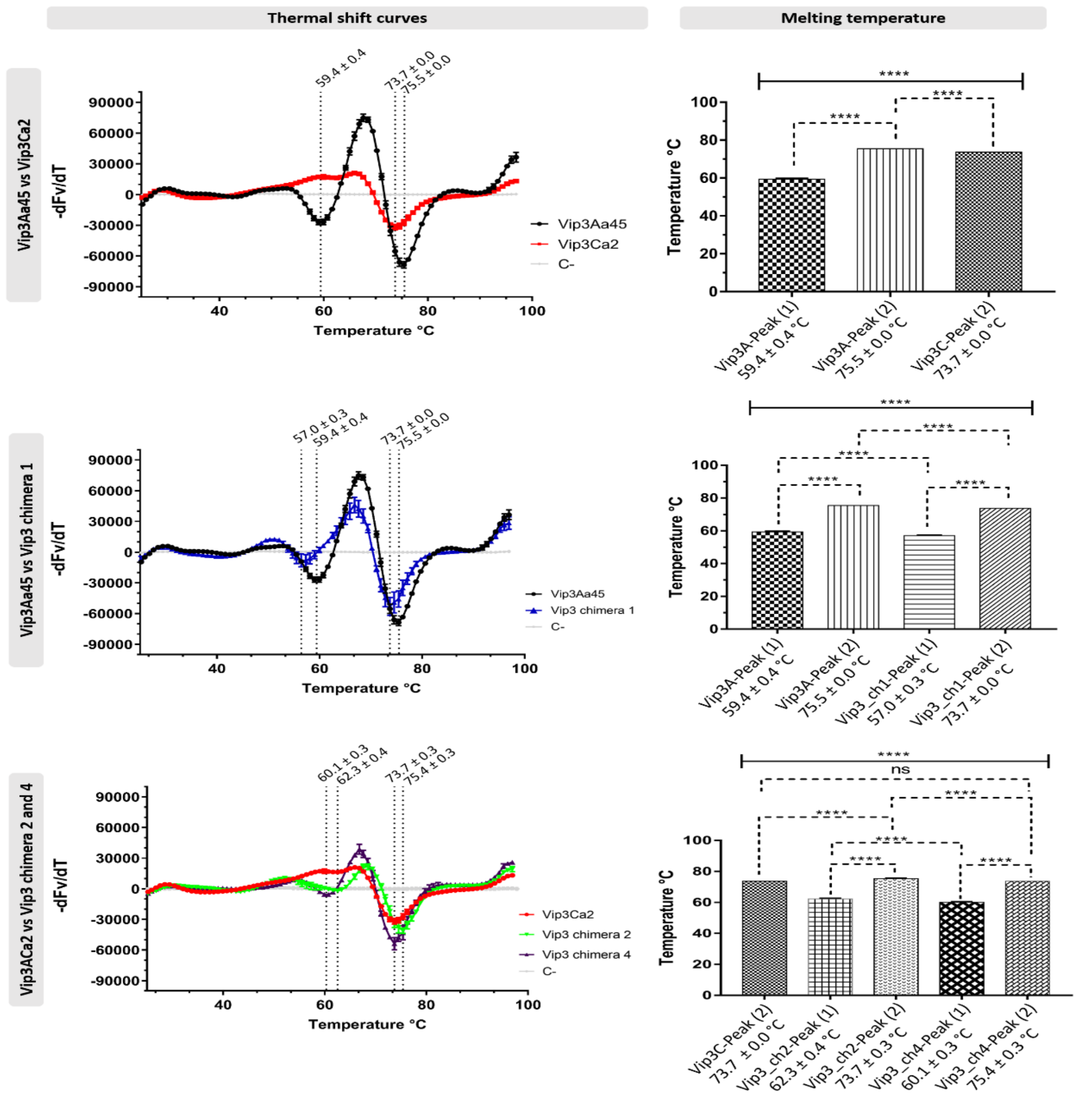

Figure S1. Thermal shift assays and multiple comparison of the thermal transitions of the parental proteins and chimeric proteins. The dashed vertical lines in the thermal shift assays curves indicate the Tm (measured in Celsius degrees) of respective thermal transitions. C- indicate the fluorescence intensity due to the SPYRO-Orange 15X in 20 mM Tris 500 mM NaCl pH 8.6. Thick line indicate the comparison of the Tm by One-way Anova (α 0.05). Dashed line indicate the multiple comparison analyzed by Tukey's range test (α 0.05). "****" indicate a p value less than 0.0001 and "ns" indicate a p value greater than 0.05

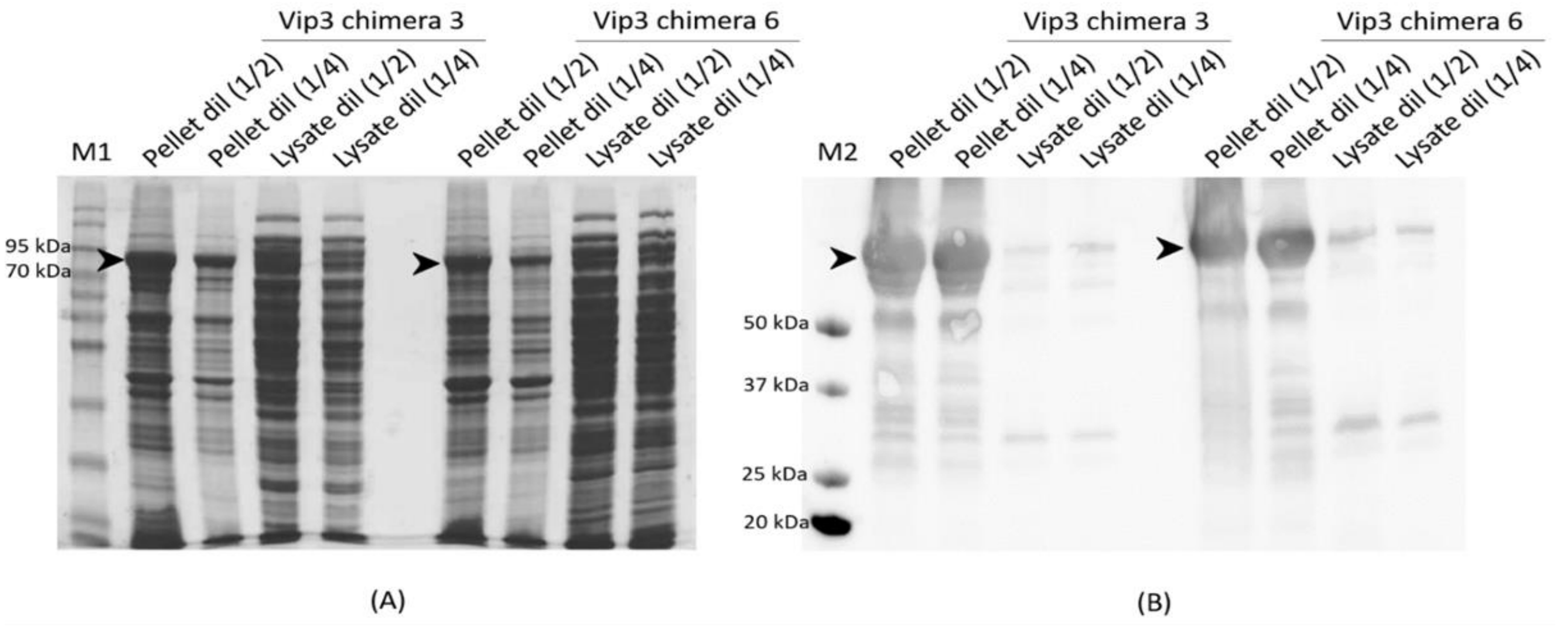

Figure S2. Expression of the chimeric Vip3 proteins (Vip3_ch3 and Vip3_ch6). (**A**) SDS-PAGE gel of different dilutions of the pellet and supernatant of the Vip3_ch3 and Vip3_ch6 proteins. (**B**) Western blot analysis different dilutions of the pellet and supernatant of the respective chimeric Vip3 proteins. The dilutions of the lysates were made with 50 mM phosphate buffer, 500 mM NaCl pH 8.0 while the pellet were dissolved in the same volume of the supernatant and the dilutions were made with 50 mM phosphate buffer, 500 mM NaCl pH 8.0. The arrowhead indicate the protein band corresponding to the chimeric Vip3 proteins. M1: Molecular Mass Marker "PINK Plus Prestained Protein Ladder" (Genedirex). M2: Molecular Mass Marker "Precision Plus Protein™ Dual Color Standards" (Biorad) developed with "Precision Protein™ Strep Tactin-HRP conjugate.

Table S1. Comparison analyses of the respective dose-response assays (LC values) of the parental and Vip3 chimeric proteins in *S. frugiperda* and *O. furnacalis*.

**Statistical variables**	***Spodoptera frugiperda*** **Vip3Aa45 vs Vip3ch2**	**Vip3Ca2 vs Vip3ch2**	***Ostrinia furnacalis*** **Vip3Ca2 vs Vip3ch4**	**Vip3ch2 vs Vip3ch4**
**Null hypothesis**	LogEC50 same for all data sets	LogEC50 same for all data sets	LogEC50 same for all data sets	LogEC50 same for all data sets
**Alternative hypothesis**	LogEC50 different from each sets	LogEC50 different from each sets	LogEC50 different from each sets	LogEC50 different from each sets
**P value**	0.22	< 0.0001	< 0.0001	< 0.0001
**F (DFn, DFd)**	1.561 (1, 35)	110.4 (1, 35)	294.2 (1, 36)	49.1 (1, 36)
**Conclusion (α= 0.05)**	Do not reject null hyphothesis	Reject null hyphothesis	Reject null hyphothesis	Reject null hyphothesis
**Preferred model**	LogEC50 same for all data sets	LogEC50 different from each sets	LogEC50 different from each sets	LogEC50 different from each sets

Table S2. Construction of the chimeric Vip3 proteins from the Vip3Aa and Vip3Ca proteins.

**Chimeric Genes**	**Individual DNA regions**								**Full ORF of *vip3* Chimeric Genes**		
	**DNA Amplicon *A***				**DNA Amplicon *B***				**DNA Amplicon *C***		
	DNA source	Size (bp)	Forward primer	Reverse primer	DNA source	Size (bp)	Forward primer	Reverse primer	DNA	Forward primer	Reverse primer
vip3 chimera 1	*vip3Ca2*	610	3	6	*vip3Aa45*	1836	5	2	*A+B*	3	2
vip3 chimera 2	*vip3Aa45*	610	1	8	*vip3Ca2*	1880	7	4	*A+B*	1	4
vip3 chimera 5	*vip3Aa45*	1573	1	10	*vip3Ca2*	895	9	4	*A+B*	1	4
vip3 chimera 6	*vip3Ca2*	1594	3	12	*vip3Aa45*	874	11	2	*A+B*	3	2
vip3 chimera 3	*vip3 chimera 5*	610	1	8	*vip3 chimera 6*	1836	7	2	*A+B*	1	2
vip3 chimera 4	*vip3 chimera 6*	610	3	6	*vip3 chimera 5*	1880	5	2	*A+B*	3	4

Table S3. Primers used in construction and sequencing of the genes encoding the chimeric Vip3 proteins.

Primers	Sequences* (5′→3′)	Source
	Overlapping PCR	
	End primers	
**[1]** Vip3Aa45 FXA/BamHI	*cgcggatcc*atcgaaggtcgtatgaacaagaataatactaaat	Designed in this study
**[2]** Vip3Aa45 FXA/NotI	*aaggaaaaaagcggccgc*ttacttaatagagacatcgtaa	Designed in this study
**[3]** Vip3Ca2 FXA/BamHI	*cgcggatcc*atcgaaggtcgtatgaacatgaataatactaaat	Designed in this study
**[4]** Vip3Ca2 Fxa/NotI	*aaggaaaaaagcggccgc*ttattcaatcttttccttaata	Designed in this study
	Annealing primers	
**[5]** ch1 20Ca-66Aa_F	gaaatttgataaattaacatttgctacagaaactagttcaaaag	Designed in this study
**[6]** ch1 20Ca-66Aa_R	cttttgaactagtttctgtagcaaatgttaatttatcaaatttc	Designed in this study
**[7]** ch2 20Aa-66Ca_F	gaaaaatttgaggaattaacttttgccacagaaagcactctaagag	Designed in this study
**[8]** ch2 20Aa-66Ca_R	ctcttagagtgctttctgtggcaaaagttaattcctcaaatttttc	Designed in this study
**[9]** ch5 20-30Aa-33Ca_F	gattaattactttaacatgtaaatcttacctgcgagaatatttattag	Designed in this study
**[10]** ch5 20-36Aa-33Ca_R	ctaataaatattctcgcaggtaagatttacatgttaaagtaattaatc	Designed in this study
**[11]** ch6 20-33Ca-33Aa_F	cattaaccttaaaatgtaaatcatatttaagagaactactgc	Designed in this study
**[12]** ch6 20-33Ca-33Aa_R	gcagtagttctcttaaatatgatttacattttaaggttaatg	Designed in this study
	Sequencing	
T7 promoter	taatacgactcactatag	pet system manual
T7 terminator	gctagttattgctcagcgg	pet system manual
Sp6 promoter	atttaggtgacactatag	pgem-t easy manual
M13 Forward (−20)	gtaaaacgacggccag	pcrtopo2.1 manual
M13 reverse	caggaaacagctatgac	pcrtopo2.1 manual
Vip3 internal 1	gatgtaatgaaacaaaattatgc	Designed in this study
Vip3 internal 2	ctaaaacaaaattatcaagtcg	Designed in this study

* The italic and underlined nucleotides indicate the extra base pairs need it to cut the NotI and BamHI close to the edge of the fragment. The italic nucleotides shown the NotI and BamHI restriction sites. The underlined nucleotides are the recognition sequence for the FXA protease. The nucleotides in red are the part of the primer in the overlapping PCR corresponding to the Vip3Ca protein.
